# Family Medicine Research Productivity in Saudi Arabia for 15 Years: An Urgent Call for Action

**DOI:** 10.7759/cureus.5955

**Published:** 2019-10-21

**Authors:** Amani A Alharbi, Ohoud A Alharbi, Zainab A Alkhayat, Lara M Arafsha, Yara M Arafsha

**Affiliations:** 1 Family Medicine, King Abdulaziz University Hospital, Jeddah, SAU; 2 Obstetrics and Gynecology, King Abdulaziz Hospital, Jeddah, SAU

**Keywords:** family medicine, research productivity, saudi arabia

## Abstract

Family medicine is one of the most vital health specialties in the field of medicine. This can be attributed to the wide range of health services for all people regardless of age, gender, and diagnosis. Saudi Arabia as suggested by various studies put more of its attention at all levels to family medicine in order to produce an adequate number of family physicians and improve both the academic aspects and the services provided by family medicine in the country. Thus, a comprehensive national survey was suggested to analyze the current situation of the specialty in the country and to draw up a strategic plan to achieve the national vision for family medicine by 2020. In light of the aforementioned, this study deemed it necessary to examine family medicine research productivity in Saudi Arabia in order to recommend possible measures to increase the quality of research output related to the field. The Web of Science (WoS) bibliographic database search engine was used to retrieve and analyze data. The terms “Family Medicine” and “Saudi Arabia” were entered in the search address fields using the SAME boolean operator (i.e., Family Medicine SAME Saudi Arabia) to retrieve records that contained these two terms and were in the same address. Research articles that were published from 1 January 2004 to 31 December 2018 were included for data analysis. Publication details such as the year of publication, document type, research area, authors’ affiliation, journal name, international collaborators, journal impact factor (JIF), and citation reports were all considered in the analysis. As for the result, Saudi Arabia’s research productivity in the field of family medicine is very poor, with a very gradual linear increase over the last 15 years. Further, health institutions have low research productivity compared to universities and medical colleges. Finally, most of the publications were published in low- or no-impact-factor journals. Therefore, this study concludes that Saudi Arabia has had low research productivity in family medicine. It recommends that an educational research program with the supervision of Ministry of Health (MOH) be implemented among family medicine physicians featuring organizational support and well-structured communication between health and educational institutions to increase research productivity in the country.

## Introduction and background

Although family medicine is still an emerging specialty that is struggling to find its niche in the medical profession worldwide, there is no denying that it is one of the most vital fields of medicine. This can be attributed to the wide range of health services that it provides to all people regardless of age, gender, and affected organ or system. Family medicine is supported by a few important principles: comprehensiveness, continuity, coordination, and accessibility. The United States of America (USA) recognized this specialty in 1969 when it created the 20th medical specialty board for the field [1].

Even though Middle Eastern culture is known for its solid family network, family medicine in Saudi Arabia has grown gradually and became known medical specialty in Arab nations [2]. Recognition of the specialty began in 1978 with the Alma Ata Declaration. It was issued by the World Health Organization General Assembly when the country recognized improving primary health care (PHC), one of the significant systems for delivering high-quality health services [3]. Saudi Arabia advanced and adjusted the idea of PHC in 1983, using it as the basis for its healthcare system [4]; this later led to an upgraded delivery of services [5].

The advancements in the field of family medicine did not happen without challenges, yet the country has recognized its importance despite the numerous challenges practitioners encountered. Numerous studies have highlighted the benefits and impact of primary care and family physicians [6].

Healthcare research plays a pivotal role in raising the bar of excellence in healthcare delivery [7]. The study on the subject can provide a clear picture of the extent of the problem and even determine the result of interventions utilized for different medical problems [8]. The findings can also serve as the basis to aid in decision-making or even policy-making in coming up with evidence-based guidelines about the soundest approaches to certain health problems [9-11].

In the past few decades, many studies have been conducted on PHC in various developed countries; however, the research differs tremendously from one country to the next [12]. As practitioners have paid more attention to evidence-based practice in primary care in the Saudi Arabian context, they have encountered specific challenges and obstacles such as the lack of opportunities for professional development in PHC [11].

The literature search revealed a dearth of studies focusing on evaluating biomedical research in Saudi Arabia [8-9, 13-14]. One such undertaking was a study by Al-Ahmadi and Roland [3] that aimed to retrieve and record journal articles focusing on PHC in Saudi Arabia.

In contrast, some studies from Saudi Arabia about family medicine report that more attention is required at all levels of family medicine in order to produce an adequate number of family physicians and improve both the academic aspects and the services provided by family medicine in the country [7, 15-19]. Overall, there is a need for a comprehensive national survey to analyze the current situation of the specialty in Saudi Arabia and to draw up a strategic plan to achieve the national vision for family medicine by 2020 [2].

In light of the aforementioned, this researcher deemed it necessary to examine family medicine research productivity in Saudi Arabia in order to recommend possible measures to increase the quality of research output related to the field. 

## Review

Methods

This review provides information about family medicine research productivity in Saudi Arabia. The data were sourced from the Web of Science (WoS) Thompson Reuters International Scientific Indexing (ISI) bibliographic database. The research articles were retrieved by entering “Family Medicine SAME Saudi Arabia” in the search address field. The use of the SAME boolean operator specified that the terms “Family Medicine” and “Saudi Arabia” would be in the same address, ensuring the search engine retrieved records that contained these two terms. The search was performed on 30 May 2019 to maximize the search results. This was also done to include research articles published in certain biomedical journals for the month of December 2018, which typically appear in the WoS index within 2-14 days (or rarely, more than two months) after the journal issue is submitted for indexing.

Of the 467 research articles dated from 1 January 2004 to 31 December 2018 based on the search results, 415 (88.86%) articles were included in the data analysis. Letters, editorial papers, abstracts, meetings, conferences, corrections, reference materials, books, and unspecified items were excluded. The family medicine research in terms of its publication details such as the year of publication, document type, research areas, authors’ affiliation, journal name, international collaborators, and journal impact factor (JIF) are presented by frequency and percentage below.

Results

According to Year of Publication

**Figure 1:**
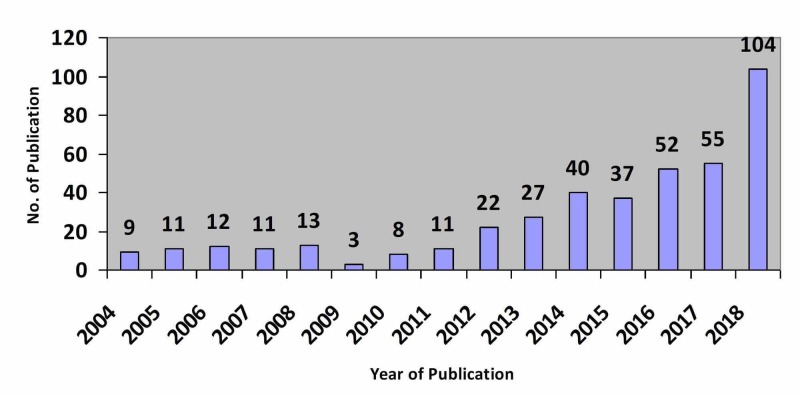
Frequency of family medicine research publication according to year.

Figure 1 illustrates that Saudi Arabia’s research productivity in the field of family medicine is very poor, presenting a slow linear increase for the 15 years studied. The majority of the articles were published in 2018 (104, 25.06%), followed by 2017 (55, 13.25%), and 2016 (52, 12.53%). There was a slight downward trend in the end of 2015 (37, 8.91%). It is worth noting that the lowest number of publications was in 2009 (3, 0.72%).

According to Document Type

Most of the publications were research articles (399, 96.14%), followed by systematic reviews and meta-analyses (11, 2.65%), case reports (4, 0.96%), and clinical trials (1, 0.24%).

According to Research Area

The majority of the studies were related to general internal medicine (32.53%), followed by public environmental/occupational health (7.47%), and then healthcare sciences services (6.26%). Those focusing on pharmacology/pharmacy ranked next (5.54%). Those pertaining to endocrinology/metabolism and education/educational research were almost of the same number (3.85% and 3.61%, respectively). Topics about infectious diseases scored the lowest (2.41%). Finally, 30.12% of the publications comprised research areas that had a frequency of less than 10. All of these data are shown in Table 1.

**Table 1:** Frequency of family medicine research publications according to research area.

Top 10 research areas	Frequency	Percentage (N = 415)
General internal medicine	135	32.53
Public environmental/occupational health	31	7.47
Health care sciences services	26	6.26
Pharmacology/pharmacy	23	5.54
Endocrinology/metabolism	16	3.85
Education/educational research	15	3.61
Biochemistry/molecular biology	12	2.89
Research/experimental medicine	11	2.65
Respiratory system	11	2.65
Infectious diseases	10	2.41
Others	125	30.12
Total	415	100.00

According to Institution/Affiliation

Table 2 shows that majority of the proponents of the publications considered in this study were from King Saud University (18.07%), followed by those from the Ministry of National Guard Health Affairs (MNGHA) at 14.70%. A total of 9.64% of the authors were affiliated with King Faisal Specialist Hospital and Research Center (KFSHRC), and 8.43% of the authors were from the Ministry of Health (MOH). The rest came from several governmental universities and hospitals. Finally, 28.92% of the publications combined were from different institutions. These were the institutions published with less than 10 research articles.

**Table 2:** Frequency of family medicine research publications according to institution/affiliation.

Top 10 institutions/authors’ affiliation	Frequency (N = 415)	Percentage
King Saud University	75	18.07
Ministry of National Guard Health Affairs (MNGHA)	61	14.70
King Faisal Specialist Hospital and Research Center (KFSHRC)	40	9.64
Ministry of Health (MOH)	35	8.43
King Abdulaziz University	17	4.10
King Khalid University	17	4.10
Al Majmaah University	16	3.86
Prince Sattam Bin Abdulaziz University	14	3.37
King Fahad Medical City	10	2.41
Prince Sultan Military and Medical City	10	2.41
Other	120	28.92
Total	415	100.00

According to Journal of Publication

At the top of the list is the Saudi Medical Journal (10.60%), followed by the Journal of Family and Community Medicine (JFCM) (8.91%), then the Journal of Family Medicine and Primary Care (5.54%). There was only 3.61% of the articles published in the Annals of Saudi Medicine. Those published in the International Journal of Health Sciences and World Family Medicine had a similarly low frequency (2.41%). The rest of the studies were published in the International Journal of Health Sciences (IJHS), the Pakistan Journal of Medical Health Sciences, the Pakistan Journal of Medical Sciences, PLOS ONE, Electronic Physician, Medical Teacher, and Saudi Journal of Kidney Diseases and Transplantation (see Table 3).

**Table 3:** Frequency of family medicine research publications according to journal.

Top 10 journals of publication	Frequency (N = 415)	Percentage
Saudi Medical Journal	44	10.60
Journal of Family and Community Medicine	37	8.91
Journal of Family Medicine and Primary Care	23	5.54
Annals of Saudi Medicine	15	3.61
International Journal of Health Sciences	10	2.41
World Family Medicine	10	2.41
International Journal of Health Sciences (IJHS)	6	1.44
Pakistan Journal of Medical Health Sciences	6	1.44
PLOS One	6	1.44
Electronic Physician	5	1.20
Medical Teacher	5	1.20
Saudi Journal of Kidney Diseases and Transplantation	5	1.20
Others (i.e., less than five articles)	243	58.55
Total	415	100.00

According to International Collaborator

Table 4 illustrates that 139 (33.49%) out of the 415 research articles analyzed were mostly published as collaborations (10 different countries). The collaborators were mainly from Egypt (9.40%), followed by Canada, the USA, and Pakistan (4.58%, 4.34%, and 3.86%, respectively). The smaller numbers of collaborations came from England, the United Arab Emirates, Malaysia, the Netherlands, Finland, and India.

**Table 4:** Frequency of family medicine research publications according to international collaborators.

Top 10 international collaborators	Frequency (N = 139/415)	Percentage
Egypt	39	9.40
Canada	19	4.58
USA	18	4.34
Pakistan	16	3.86
England	13	3.13
United Arab Emirates	8	1.93
Malaysia	7	1.69
Netherlands	7	1.69
Finland	6	1.45
India	6	1.45
Total	139	33.49

According to Journal Impact Factor (JIF)

Table 5 shows that 51.08% of the publications were published in a journal with a JIF of zero. The highest number of publications (19.28%) was published in journals with 1.00-1.99 JIF, whereas the lowest (0.24%) was published in journals with either 6.00-6.99 or 7.00-8.99 JIF. Surprisingly, there were four articles (0.96%) published in journals with high JIFs of 25.00-50.00 (i.e., Lancet journals).

**Table 5:** Frequency of family medicine research publications according to journal impact factor.

Journal impact factor	Frequency (N = 415)	Percentage
0.01–0.99	52	12.53
1.00–1.99	80	19.28
2.00–2.99	48	11.57
3.00–3.99	14	3.37
4.00–5.99	3	0.72
6.00–6.99	1	0.24
7.00–8.99	1	0.24
25.00–50.00	4	0.96
Unspecified	212	51.08
Total	415	100

According to Citation Report

The 415 publications on family medicine research from Saudi Arabia in January 2004 to December 2018 had a Hirsch index (h-index) of 21. There was an average of 6.94 citations per publication. There was a total of 2,879 citations, and 2,817 of these did not have any self-citations.

Discussion

Family medicine is a unique specialty, with high emphasis on family, health promotion, and disease prevention. Physicians should have broad exposure to the health care of all age groups as well as substantial experience in managing diverse pathological conditions. This includes experience in those conditions that are commonly encountered in primary care practices, featuring a wide range of acute and chronic medical conditions in ambulatory settings [20]. This explains why the research areas in this field are broad enough to cover everything in the health continuum of family medicine practice, a claim further validated by the findings of this study and other studies done in Saudi Arabia and other parts of the world [2, 21-24].

This review revealed almost the same results as those by Jahan and Al-Saigul [21] in terms of a linear growth trend in research productivity over time, a majority of original research articles being the main type of publication, and that most of the authors were from universities. However, this study dealt with publications from 2004 to 2018, whereas Jahan and Al-Saigul [21] included research from 1983 to 2011. Also, this study included variables like journals of publication, international collaborators, JIF, and citation reports; Jahan and Al-Saigul did not include these variables in their study.

Aside from instruction and community engagement, research is one of the tri-fold functions of universities. Expectedly, the majority of the biomedical publications were produced from work conducted by universities or medical colleges [12-13, 21]. A study from Turkey reported similar findings that almost all the family medicine publications (99%) were from universities [12]. However, another study from Saudi Arabia reported that just over half (54.6%) of all biomedical publications were authored by educators [13]. Jahan and Al-Saigul [21] yielded the same findings, which can be attributed to the fact that medical professionals-cum-educators are required to publish papers, and that their universities often have family medicine and community medicine departments.

Among the Gulf Cooperation Council (GCC) countries, Saudi Arabia has increased its research productivity and citations; it also holds the record for the highest h-index value in the region, which was driven by the government’s focus on prioritizing research and education [25]. In fact from 1996 to 2012, Saudi Arabia produced 16,196 research papers in medicine, 14,732 citable documents, 102,827 total citations, 6.36 citations per document, and had an h-index of 92. However, if all the medical and allied health sciences research publications are summed, they yield a total of 27,246 research papers, 25,416 citable documents, 181,999 total citations, 7.07 mean citations per document, and a mean h-index of 41.44 [8].

In contrast, Saudi Arabia’s family medicine and PHC research outputs are relatively low, even though the PHC setup in Saudi Arabia centrally run by MOH. Likewise, the majority of the outputs were cross-sectional studies and were authored by educators. This fact is a clear call to action to enhance PHC research to create a supportive environment in bringing about an increased evidence base for PHC, leading to the effective delivery of health services [26].

This review revealed that the highest proportion (10.60%) of articles analyzed were published in the Saudi Medical Journal, which also corresponds to Mesawa et al.’s [27] findings, although to a higher extent (they reported 23.5%). Conversely, this proportion was more than halved in terms of publication in the JFCM.

It is worth noting that the Saudi Medical Journal was founded in 1979 and has been a monthly publication since 1999; hence, is the oldest medical journal in Saudi Arabia [26]. In contrast, the JFCM was established in late 1994 and was published once every six months until the year 2000, after which it increased its publication to once every four months [28]. It can thus be concluded that although the JFCM focuses more on PHC and community-based research, the Saudi Medical Journal still contains the majority of the research publications because it is the pioneer of the field in terms of its founding date.

One novel aspect of this study in comparison with the others is its attempt to perform a quantitative analysis of the research impact of family medicine publications in terms of their JIFs and h-indexes. Two approaches can be used to measure research impact: quantitative or qualitative. Citation counts, the h-index, and JIF are quantitative measurements. However, the qualitative description of research impact does not feature a singular current tool or system that completely measures it. There are many ways to measure scholarly impact on a field or discipline. Traditionally, the measurement of research impact was determined by the number of times other researchers cited an author’s article. Although it is easy to create a scholarly work publication list and indicate the occasions when they have been referred to by other authors, various calculations of an author’s research production have also been created [29].

This review revealed that more than half of the studies (51.08%) were published in a journal with an unspecified JIF; 19.28% were published in journals with JIF of 1.00-1.99, whereas only 0.96% was published in journals with high JIF (25.00-50.00). In a similar study by Latif examining 2008-2012, a detailed analysis of the JIF of biomedical research from Saudi Arabia revealed that only a small fraction of papers (4, 0.26%) appeared in journals with a high JIF (≥7) as listed by JCR 2012 Science Edition: Lancet (39.06), Gastroenterology (18.82), Circulation (15), and Acta Neuropathology (9.7) [13]. In the present review, the majority of the publications were in journals with unspecified or low JIFs, whereas only a small fraction were published in those with high JIFs.

The JIF is the most well-known metric for assessing journal performance or quality. The JIF can be calculated after a journal has been published for a minimum of three years; thus, JIFs cannot be calculated for new journals. Journals with the highest JIF are those that publish the most commonly cited articles over a two-year period. The JIF applies only to journals -- not to individual articles or individual scientists, unlike the h-index. The h-index is an author-level research metric to measure authors’ research productivity and their publications’ citation impact. Being such, it is both a qualitative and quantitative measure of authors’ research outputs. It focuses more specifically on the impact of individual scholars instead of an entire journal. It can be said that the higher the h-index, the more scholarly output a researcher has [30]. But over time, a range of different research metrics have become available. In spite of the increasing research metrics available, the present researcher only considered JIF and h-index. However, researchers have opposing views about treating JIF as a metric of the quality of publication or as it cannot fully capture the true relevance of the individual article.

## Conclusions

Despite the country’s noticeable research achievements in the field of medicine in general, Saudi Arabia’s family medicine research productivity during the 15-year period studied here was poor. However, the two-fold increase in research publications in 2018 could be a significant sign of research productivity improvement in the country. This review revealed that the research that was published was mostly observational studies; thus, analytical and experimental studies must also be done. Lastly, research educational programs with the supervision of the MOH featuring organizational support, and well-structured communication between health and educational institutions are all needed to increase research productivity in both types of institution. The findings of this review are limited to research articles indexed in WoS; those published in predatory or pseudo journals were not included.
